# Molecular characterization of cancer-intrinsic immune evasion genes indicates prognosis and tumour microenvironment infiltration in osteosarcoma

**DOI:** 10.18632/aging.205074

**Published:** 2023-10-04

**Authors:** Xiaokun Zhao, Jian Zhang, Jiahao Liu, Shengzhong Luo, Rui Ding, Xinxin Miao, Tianlong Wu, Jingyu Jia, Xigao Cheng

**Affiliations:** 1 Department of Orthopedics, The Second Affiliated Hospital of Nanchang University, Nanchang, Jiangxi 330006, China; 2 Institute of Orthopedics of Jiangxi, Nanchang, Jiangxi 330006, China; 3 Institute of Minimally Invasive Orthopedics, Nanchang University, Nanchang, Jiangxi 330006, China

**Keywords:** osteosarcoma, cancer-intrinsic immune evasion, prognosis, tumor microenvironment, biomarker

## Abstract

Cancer-intrinsic immune evasion (IE) to cells is a critical factor in tumour growth and progression, yet the molecular characterization of IE genes (IEGs) in osteosarcoma remains underexplored. In this study, 85 osteosarcoma patients were comprehensively analyzed based on 182 IEGs, leading to the identification of two IE clusters linked to distinct biological processes and clinical outcomes. In addition, two IE clusters demonstrated diverse immune cell infiltration patterns, with IEGcluster A displaying increased levels compared to IEGcluster B. Moreover, an IE score was identified as an independent prognostic factor and nomogram may serve as a practical tool for the individual prognostic evaluation of patients with osteosarcoma. Finally, GBP1, a potential biomarker with high expression in osteosarcoma was identified. The findings of this study highlight the presence of two IE clusters, each associated with differing patient outcomes and immune infiltration properties. The IE score may serve to assess individual patient IE characteristics, enhance comprehension of immune features, and guide more efficacious treatment approaches.

## INTRODUCTION

Osteosarcoma, a malignant bone tumor originating from mesenchymal cells, predominantly affects children and young adults [1, 2]. Although a variety of treatments such as surgery, chemotherapy, and radiation therapy have led to increased patient survival rates [3, 4], 10–25% of osteosarcoma patients still develop metastases at the time of diagnosis, with approximately 90% of these metastases presenting in the lungs [5–7]. The prognosis for individuals with advanced, metastatic, recurrent, or drug-resistant osteosarcoma remains bleak, despite the range of available medications [8, 9]. To better assess the prognosis of patients with osteosarcoma and to develop new therapeutic measurements, a deeper understanding of the mechanisms of osteosarcoma is needed here.

The development of tumors is influenced by many factors, including drug resistance and epigenetic alterations [10–12]. Notably, the immune system plays an important role in cancer development and immunotherapy [13–15]. Immune escape events allow cancer cells to evade the elimination mechanisms imposed by the host immune system. Cancer-intrinsic immune evasion (IE) denotes the process by which neoplastic cells evade detection and destruction by the host’s immune system through various strategies, thereby enabling their survival, proliferation, and resistance to immunotherapies [16, 17]. These evasion tactics allow cancer cells to multiply and spread throughout tumorigenesis, fostering resistance to immunotherapies [18, 19]. Immunotherapeutic strategies have demonstrated benefits in various cancer patients, including those with osteosarcoma [9, 20, 21]. Therefore, an improved understanding of IE patterns, along with the underlying biological mechanisms of the osteosarcoma microenvironment, is important to improve treatment outcomes.

Bioinformatics methods are widely used to find molecular changes in the occurrence and development of diseases and are an effective way to explore the pathogenesis of diseases [22–26]. The present investigation utilized transcriptional data and clinical information from 85 osteosarcoma patients to identify two unique IE clusters in osteosarcoma. A thorough evaluation was performed to understand the correlation between these clusters and immunological features. An IE score system was also established to quantify the IE patterns at an individual patient level, with validation carried out across multiple independent datasets. Finally, GBP1, a gene highly expressed in osteosarcoma, was identified as a potential prognostic biomarker with promising implications.

## MATERIALS AND METHODS

### Data source

From the TARGET-OS database, 85 osteosarcoma patients were selected for cluster analysis and used as a training group for follow-up analysis. The GSE21257 and the TCGA-SARC cohort were used as verification cohorts, which separately included 53 osteosarcoma patients and 262 sarcoma patients. GSE225588 (*n* = 12) and GSE99671 (*n* = 36) were used to validate the expression of GBP1. The IEGs were obtained from a previous study [18] and presented in Supplementary Table 1.

### Unsupervised consensus cluster analysis

The “ConsensusClusterPlus” R package was employed to conduct consensus clustering. The similarity distance was calculated using Euclidean distance, and the data were clustered using the k-means clustering method with a fixed inclusion rate of 80% of patients in each iteration for 100 iterations.

### Tumour immune microenvironment

The levels of immune infiltration were estimated through the application of single sample Gene Set Enrichment Analysis (ssGSEA) [27]. The Cell-type Identification by Estimating Relative Subsets of RNA Transcripts (CIBERSORT) was employed to determine the abundance of 22 discrete types of immune cells [28]. The ESTIMATE algorithm, implemented via the “estimation” R package, facilitated the computation of ESTIMATE scores, immunity scores, and stromal scores for osteosarcoma patients.

### GSVA and GSEA

The biological characteristics of different IEGs were investigated by using the “GSVA” R package [29]. A gene set named “c2.cp.kegg.v7.4.symbols” was used to recognize biological processes (downloaded from the Molecular Signature Database).

Gene Set Enrichment Analysis (GSEA) was used to reveal the risk signature related pathways. The dataset “c2.cp.kegg.v7.4.symbols.gmt” was employed in the analysis. Statistically significant pathways were determined by *P* < 0.05. Enrichment results were extracted to display immune-related pathways.

### Differentially expressed genes and enrichment analysis

By utilizing the “limma” package, differentially expressed genes (DEGs) between two IE clusters were identified, whereby screening criteria were set at FDR < 0.05 and logFC > 0.585. Using the “clusterProfiler” package, we analyzed Gene Ontology (GO) enrichment and KEGG pathways to understand DEGs functions.

### Determination of IE score

A univariate Cox regression analysis was performed utilizing the “survival” R package, with a significance threshold established at *P* < 0.05 to pinpoint potential prognostic determinants among DEGs. The identified genes were then evaluated through 1000 cycles of lasso regression analysis, facilitated by the “glmnet” R package. The establishment of a prognostic signature was achieved through the utilization of multivariate Cox regression analysis. The risk score for each patient was calculated using the following formula:


$$IE\;score=\sum_{i=1}^{n}\left(Coef_{i}\times x_{i}\right)$$

Here, *Coef_i_* indicates the regression coefficients of genes, while *x_i_* represents the gene’s expression level.

### Evaluation and verification of the IE score

The osteosarcoma patients were classified into high- and low-risk groups by employing the optimal cut-off value of the risk score. In order to evaluate the difference in predictability between the two groups, Kaplan-Meier curve was used. Univariate and multivariate Cox regressions were utilized to determine the independent prognostic factors associated with osteosarcoma.

### Cell source and culture

Cell lines (MG63, HOS, Saos-2, hFOB 1.19) used in the study were purchased from the Chinese Academy of Sciences Cell Bank (Shanghai, China). The medium consisted of DMEM supplemented with 1% penicillin/streptomycin and 10% fetal bovine serum. At 37°C and 5% CO2, osteosarcoma cells (MG63, HOS, Saos-2,) were maintained, while 34°C and 5% CO2 were used to culture human osteoblasts (hFOB 1.19).

### Clinical specimens

A collection of three osteosarcoma tissues, along with three corresponding adjacent normal tissues, was assembled from patients diagnosed with osteosarcoma. These patients had undergone surgical procedures at The Second Affiliated Hospital of Nanchang University. All participating osteosarcoma patients signed a written informed consent prior to entering the study and the hospital ethics committee agreed to the study.

### RNA isolation and real-time fluorescent qPCR

RNA extraction from cells was performed using TRIzol™ Reagent (Thermo Fisher Scientific, USA). RNA was reverse-transcribed into cDNA with use of the PrimeScript™ RT kit (Takara, Shiga, Japan). As an internal control, a parallel real-time PCR was performed for GAPDH. Relative quantification was performed using the 2^−ΔΔCt^ method. The gene primers are presented in Supplementary Table 2.

### Immunohistochemical staining and pan-cancer analysis of GBP1

To confirm GBP1 expression, immunohistochemical staining (Proteintech, 15303-1-AP) was carried out on paraffin-embedded sections. These sections were subsequently examined and imaged through microscopy. By using TIMER (https://cistrome.shinyapps.io/timer/) and GEPIA2 (http://gepia2.cancer-pku.cn/), the expression of GBP1 in the pan-cancer is presented.

### Statistical analysis

The statistical analysis was performed using R 4.2.0 and SPSS Statistics 22.0. Student’s *t*-tests were used to compare the two clusters, with a *P* < 0.05 showing statistical significance.

### Data availability

The datasets supporting the conclusions of this article are available in the TARGET-OS, the Gene Expression Omnibus (https://www.ncbi.nlm.nih.gov/geo/), the TCGA database (https://portal.gdc.cancer.gov/).

## RESULTS

### Identification of two IE clusters based on IEGs

**Figure 1:**
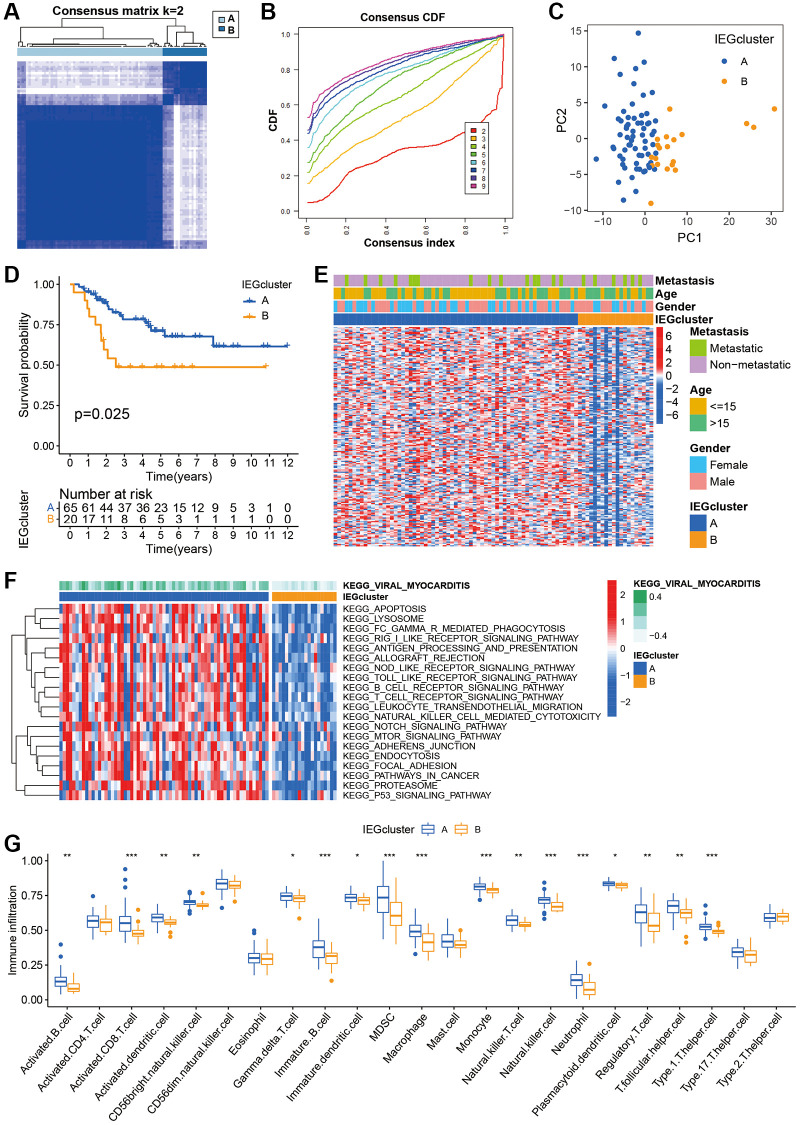
**The immune infiltration and biofunctional landscape of cancer-intrinsic immune evasion genes (IEGs) clusters.** (**A**) The consensus score matrix of 85 samples when *k* = 2. (**B**) The cumulative distribution functions (CDF) curve for *k* = 2–9. (**C**) Principal component analysis of the two clusters. (**D**) Kaplan-Meier survival analysis of the two clusters. (**E**) Heatmap of clinical characteristics of the two clusters according to the expression of IEGs. (**F**) Heatmap visualized the results of the GSVA enrichment analysis, and red represents activated pathways and blue represents inhibited pathways. (**G**) Box plot of the statistical analysis of the ssGSEA results.

Consensus clustering analysis identified two clusters (Figure 1A, 1B). Evidence of a marked difference in the dispersion between these two clusters was provided by PCA plots (Figure 1C). The prognostic analysis conducted for the two clusters revealed that IEGcluster A exhibited a remarkable survival advantage, while IEGcluster B had the worst prognosis in TARGET cohort (Figure 1D). The heatmap revealed IEGs expression and clinical characteristics in osteosarcoma, cluster A showed significantly higher expression of most of the IEGs (Figure 1E). To further probe potential disparities in the signaling pathways between the two IE subtypes, GSVA was executed. The results demonstrated that cluster A was significantly related with immune function (Figure 1F), such as NOD-like receptor signaling pathway, natural killer cell mediated cytotoxicity, and T cell receptor signaling pathway. In order to elucidate the relationship between IEGcluster and immune infiltration, ssGSEA was applied to compare the enrichment scores of immune cells in the two subtypes. A substantial discrepancy in the infiltration of most immune cells between the two IE subtypes, such as Activated CD8 T cells, and immature B cells, was noted (Figure 1G). These observations advocate that unsupervised clustering based on IEGs distribution can effectively segregate osteosarcoma into two clusters, each possessing unique clinical prognostic and tumour immunophenotypic traits.

### Comprehensive analysis of DEGs associated with IE clusters

**Figure 2:**
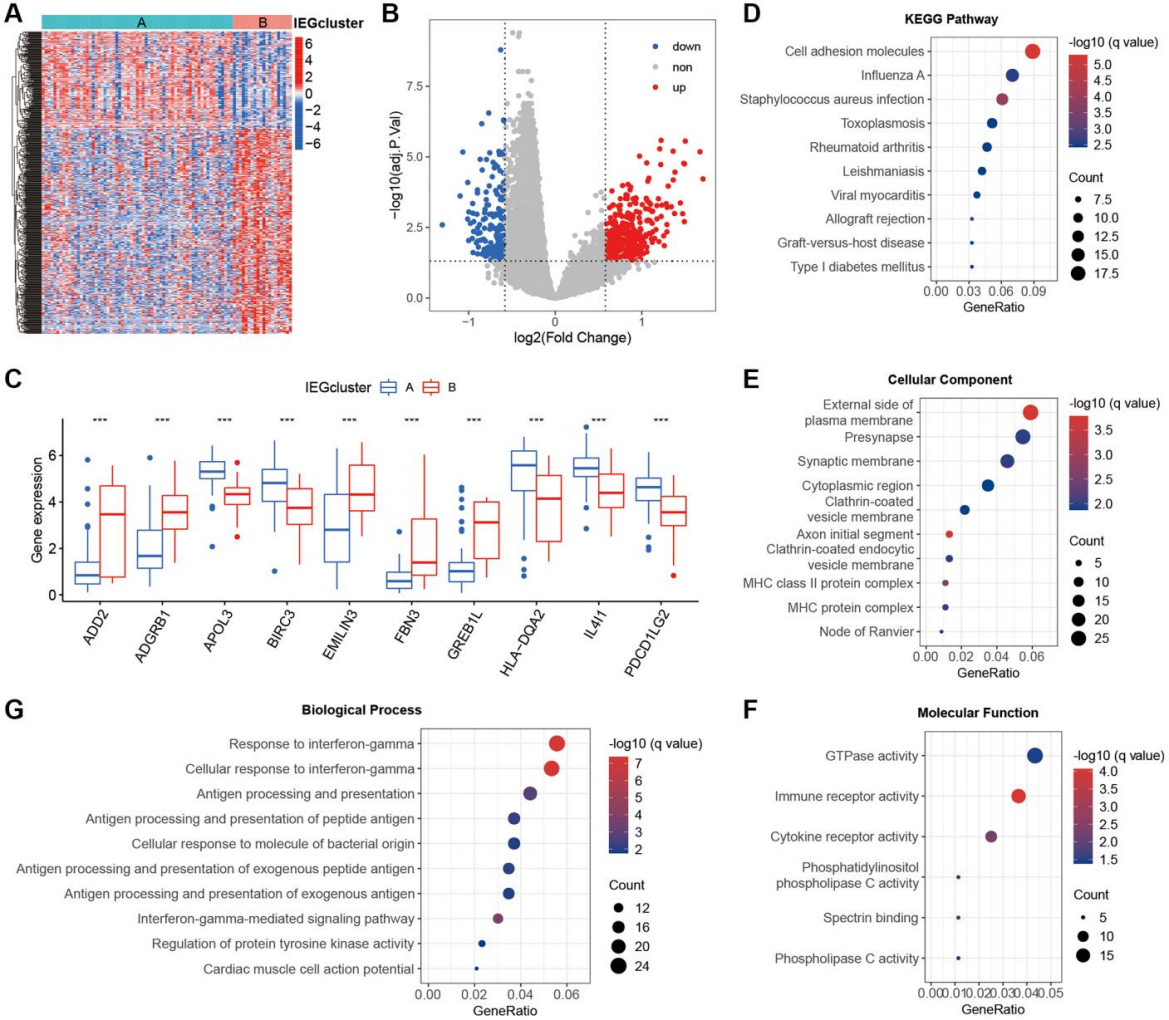
**Functional enrichment analysis between the two clusters.** (**A**) Heatmap of differentially expressed genes (DEGs) between the two clusters. (**B**) The volcano plot of DEGs. (**C**) Bubble plot of KEGG pathway analysis and (**D**–**F**) GO analysis. (**G**) Box plot showing the top 5 up-regulated and top 5 down-regulated genes.

A total of 502 DEGs were observed in IEGcluster B in comparison to IEGcluster A. These DEGs are visualized in the heatmap and volcano plot (Figure 2A, 2B). The box plot demonstrates the five most notably upregulated and downregulated DEGs (Figure 2C). Following the identification of DEGs, a functional enrichment analysis was performed, revealing a significant enrichment of immune and cancer-related pathways (Figure 2D–2G). It was suggested that IEGs greatly affects the carcinogenesis and immune microenvironment regulation of osteosarcoma.

**Figure 3:**
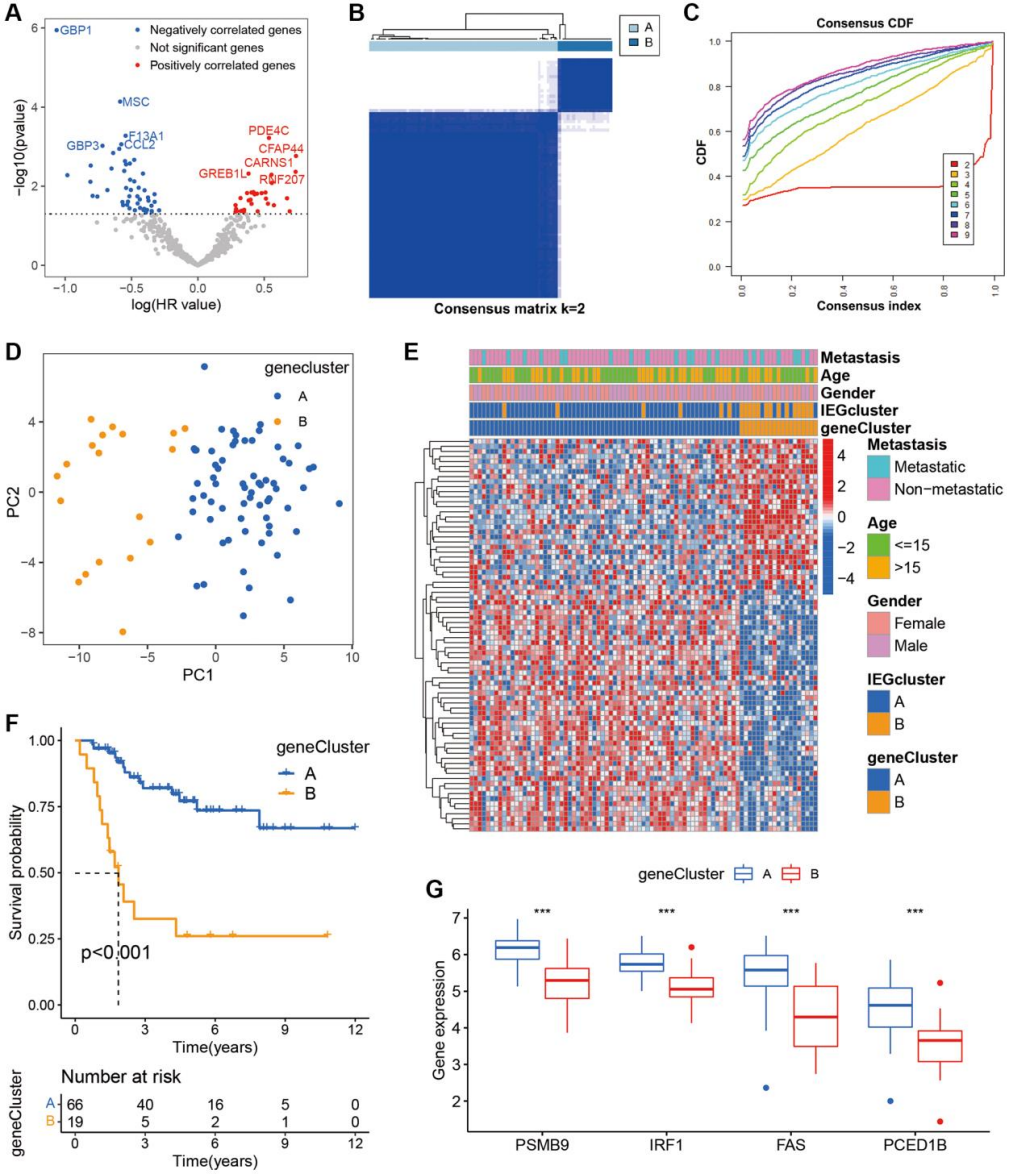
**Identification of gene clusters based on prognostic DEGs.** (**A**) Volcano plot displaying univariate Cox regression results of the 502 DEGs. (**B**) The consensus score matrix. (**C**) The CDF curve for *k* = 2–9. (**D**) PCA of the two gene clusters. (**E**) Heatmap of clinical characteristics of the two gene clusters according to the expression of prognostic DEGs. (**F**) Kaplan-Meier survival analysis of the two gene clusters. (**G**) The box plot shows genes that are significantly differentially expressed in two gene clusters.

To understand the prognostic impact of DEGs, a univariate Cox analysis was performed. The results showed that 79 DEGs affected the prognosis of osteosarcoma, with PDE4C, CFAP44, CARNS1, GREB1L, ROF207 identified as significant risk factors and GBP1, MSC, GBP3, F13A1, CCL2 as substantial protective factors (Figure 3A). Furthermore, to further explore the significance of 79 DEGs for patients, a consistent clustering algorithm was used to classify osteosarcoma patients into two gene clusters (Figure 3B, 3C). PCA plot revealed distinct distribution patterns for the two gene clusters (Figure 3D). The heatmap revealed IEGs expression and clinical characteristics in osteosarcoma, gene cluster A showed significantly higher expression of most of the IEGs (Figure 3E). Moreover, the K-M survival analysis findings revealed a statistically significant disparity in long-term survival between gene cluster A and gene cluster B (Figure 3F). At the same time, there were disparities in some IEGs between the two gene clusters (Figure 3G). These findings reinforce the reliability of the IEG-related cluster and imply an essential role of IEGs in osteosarcoma.

### Establishment and validation of IE score

**Figure 4:**
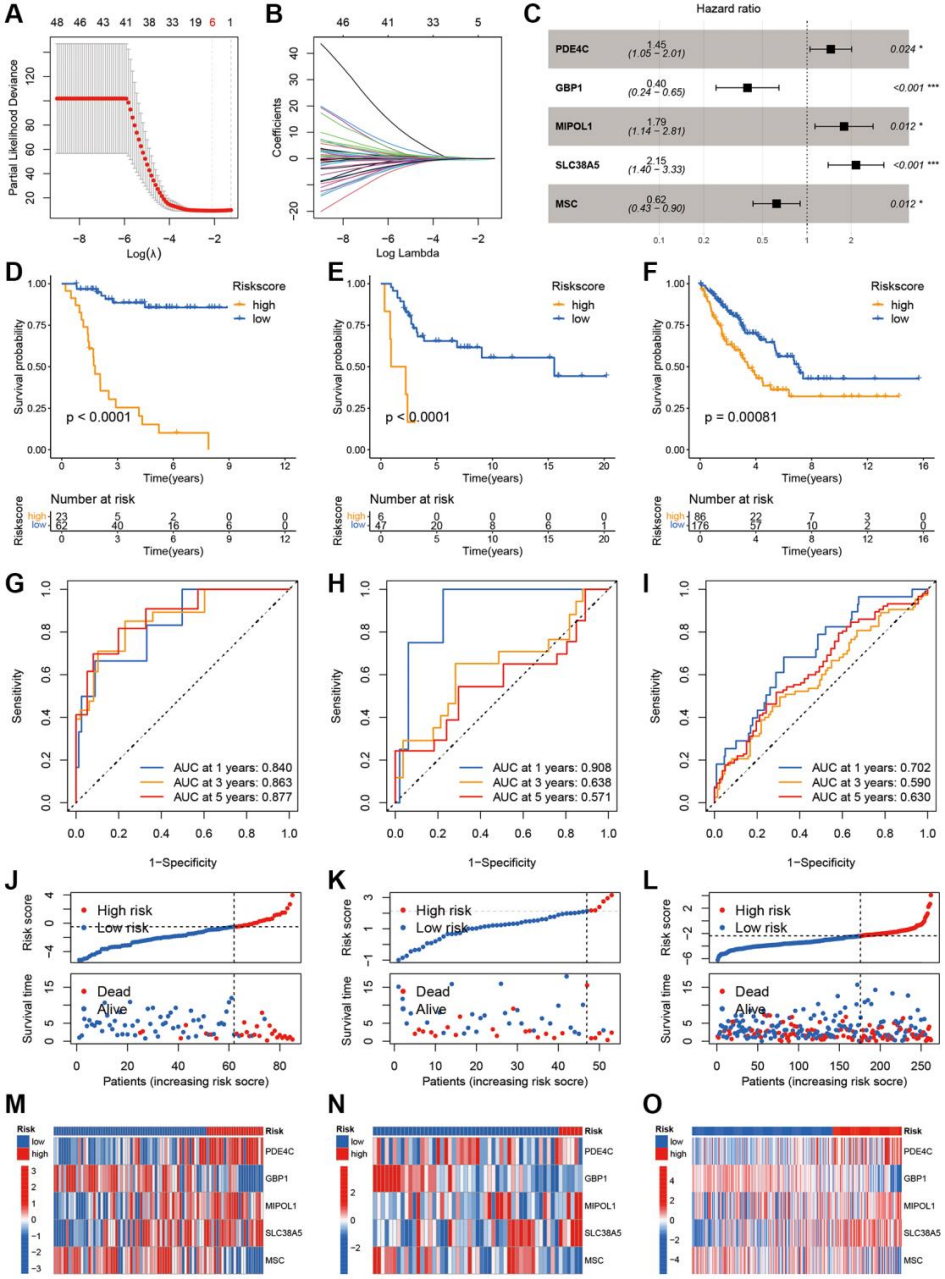
**Development and validation of the IEGs score.** (**A**, **B**) Lasso regression analysis of 79 prognostic DEGs. (**C**) Multivariate Cox regression analysis. (**D**–**F**) Kaplan-Meier curves in the TARGET, GSE21257 and TCGA-SARC cohorts. (**G**–**I**) The AUC for the prediction of 1, 3, 5 years survival rate. (**J**–**L**) Distribution of survival status and risk scores. (**M**–**O**) Heatmap of the five model genes between the high- and low-risk groups.

To formulate the IE score, seven genes were selected from the 79 IEGs based on a Lasso-Cox regression analysis (Figure 4A, 4B). Subsequently, an IE score was determined using a multivariate Cox regression analysis, and five genes and their risk coefficients were identified: PDE4C, GBP1, MIPOL1, SLC38A5, and MSC (Figure 4C). Survival analysis indicated that a higher IE score significantly foretold unfavorable outcomes across the three cohorts (Figure 4D–4F, *p* < 0.001). The ROC curve showed that the IE score was a reliable indicator of overall survival, with an area under the curve (AUC) value in all three patient groups predicting 1-year overall survival exceeding 0.7 (Figure 4G–4I). Moreover, the number of deaths gradually increased with higher IE scores; in addition, the distribution of gene expression profiles showed consistency across the three cohorts (Figure 4J–4O). These findings suggest that the developed IE score serves as a valuable prognostic indicator.

### Clinical significance of IE score

**Figure 5:**
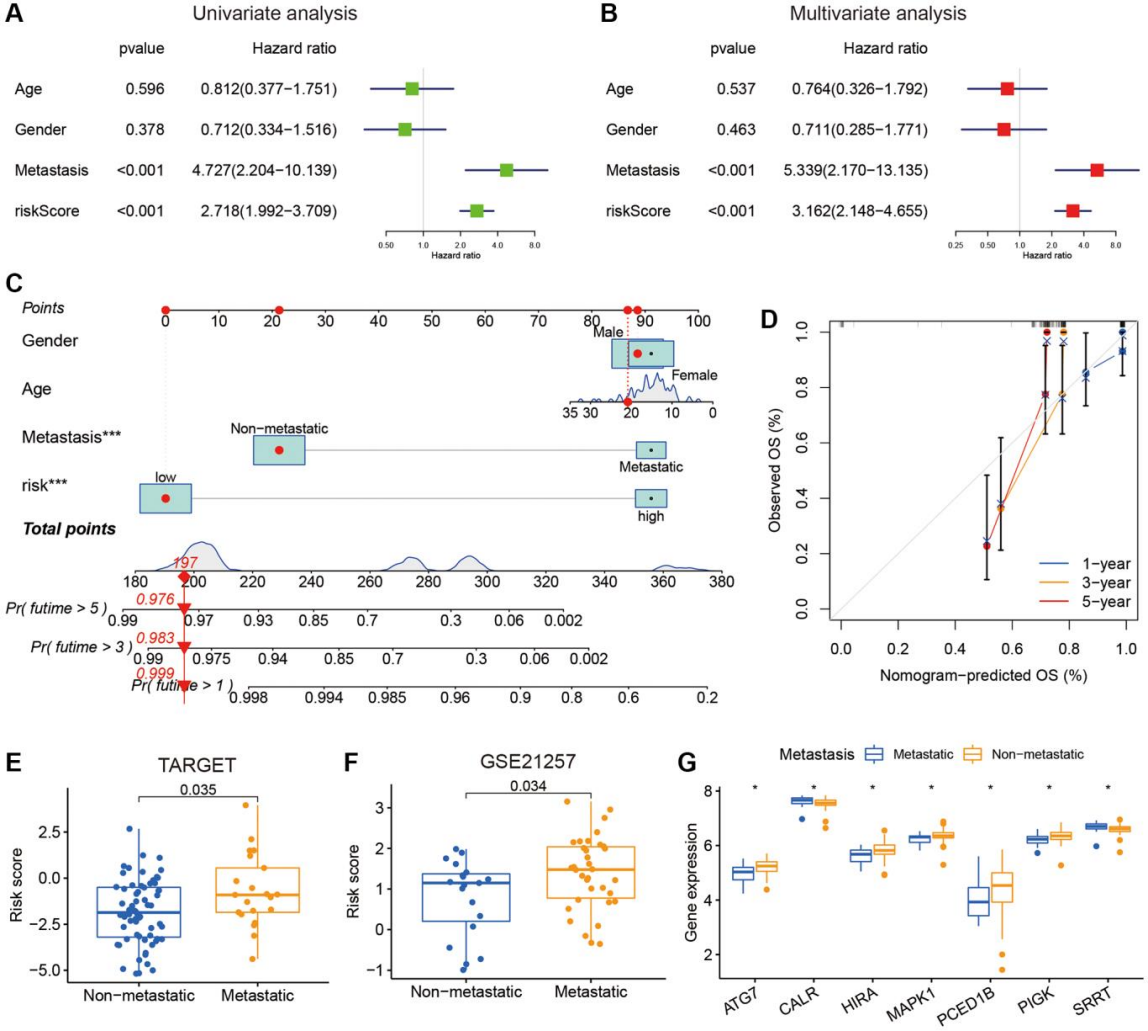
**The relationships between clinical characteristics and the IEGs signature in osteosarcoma.** (**A**, **B**) Univariate and multivariate Cox regression analysis for independent prognostic analysis of risk model. (**C**) Nomogram based on gender, age, metastasis and risk in the TARGET cohort. (**D**) The nomogram calibration curves for predicting 1-, 3-, and 5-year survival. (**E**, **F**) The relationship between the risk score and metastasis. (**G**) The expression level of the IEGs, the patients were grouped according to metastasis.

Univariate and multivariate Cox regression analyses were conducted using the TARGET dataset to assess the potential of the IE score as an independent prognostic factor. Risk score was significantly associated with OS in univariate Cox analysis (Figure 5A). The multivariate analysis also indicated the score as an autonomous prognostic indicator for osteosarcoma (Figure 5B). Subsequently, a nomogram based on scores and other clinicopathological risk factors was developed, where our risk score surfaced as the most impactful factor among multiple clinical parameters (Figure 5C). Importantly, there was an impressive agreement between predictions and actual survival rates at 1, 3 and 5 years shown by the calibration curves (Figure 5D). Recognizing osteosarcoma’s heightened malignancy and metastatic potential and the fact that distant metastasis is the primary cause of mortality in osteosarcoma patients [30], a correlation analysis was carried out between the IE score and the occurrence of metastasis. The analysis suggested that patients with metastasis in the TARGET and GSE21257 cohorts exhibited higher IE score (Figure 5E, 5F). Lastly, boxplots depicted variations in IEGs in patients with or without metastasis (Figure 5G).

### The analysis of GSEA and immune infiltration

**Figure 6:**
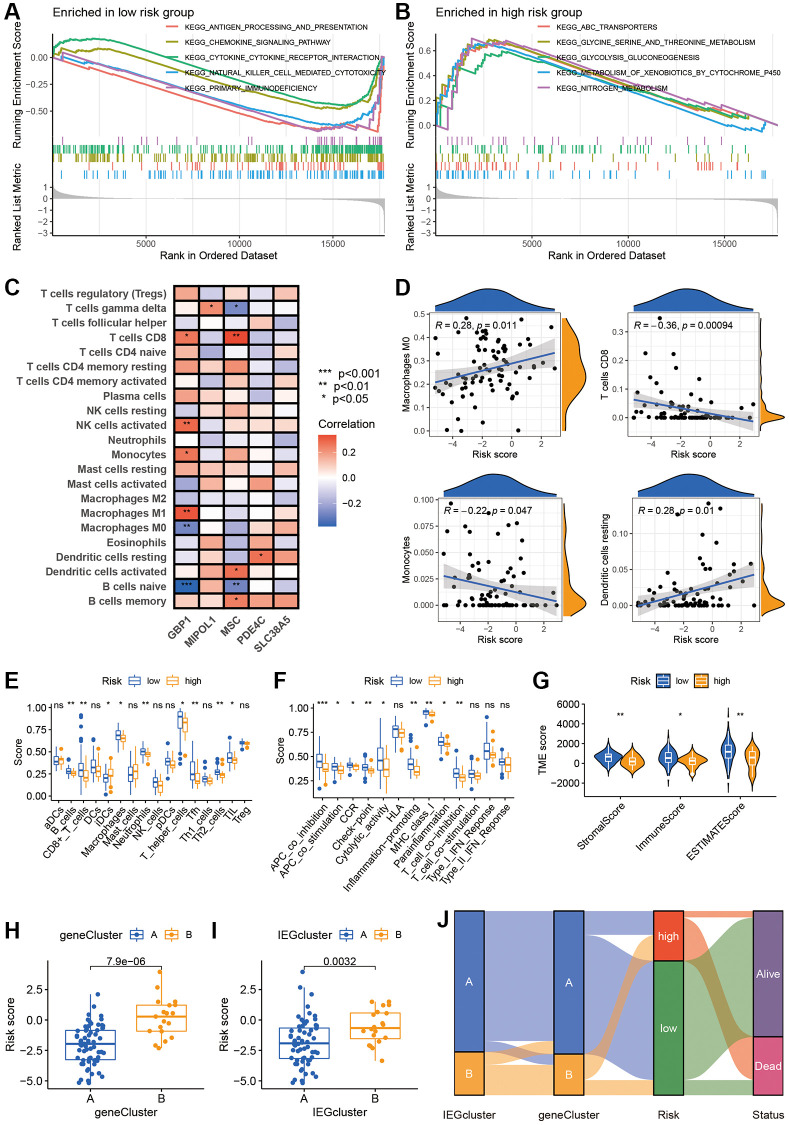
**The IEGs score was related to immune infiltration.** (**A**, **B**) GSEA was used to reveal the risk signature-related pathways. (**C**) Correlation between the five model genes and 22 immune cells based on CIBERSORT. (**D**) The association between IEGs score and immune cell infiltration. (**E**, **F**) Relationship between risk score and immune cell infiltration and related functions via ssGSEA analysis. (**G**) Risk scores were significantly correlated with Stromal scores, Immune scores, and ESTIMATE scores. (**H**, **I**) The difference in risk score among IEGs cluster and prognostic gene cluster. (**J**) Sankey plot of IEG subtype distribution in groups with different risk scores and survival status.

To assess the signaling pathways associated with IE score, GSEA analysis was performed. The analysis revealed an enrichment of “primary immunodeficiency” and “chemokine signaling pathway” in the low IE score group (Figure 6A) and an enrichment of “nitrogen metabolism” and “glycolysis gluconeogenesis” in the group with a higher IE score (Figure 6B). Subsequently, the infiltration of immune cells was examined, with heatmaps revealing a correlation between T cell CD8 and B cell naive with GBP1 and MSC (Figure 6C). Moreover, a Pearson correlation analysis demonstrated a significant positive correlation between Macrophages M0, Dendritic cells resting, and the IE score, whereas T cells CD8 and Monocytes were notably negatively correlated with the IE score (Figure 6D). Furthermore, the relationship between immune infiltration and scores was assessed based on ssGSEA. Those with high scores showed low levels of immune cell infiltration and function (Figure 6E, 6F). We also validated the appeal results using the estimates and found a considerable negative relationship between the risk scores and stromal scores, immune scores and estimated scores (Figure 6G). These findings suggest that patients with a low IE score exhibit improved prognosis and increased immune cell infiltration. The relationship between clusters and the IE score was also analyzed, with box plots indicating a high IE score in both IEGcluster B and gene cluster B (Figure 6H, 6I). The Sankey diagram illustrates the workflow in constructing the IE score (Figure 6J).

### GBP1 is highly expressed in osteosarcoma

**Figure 7:**
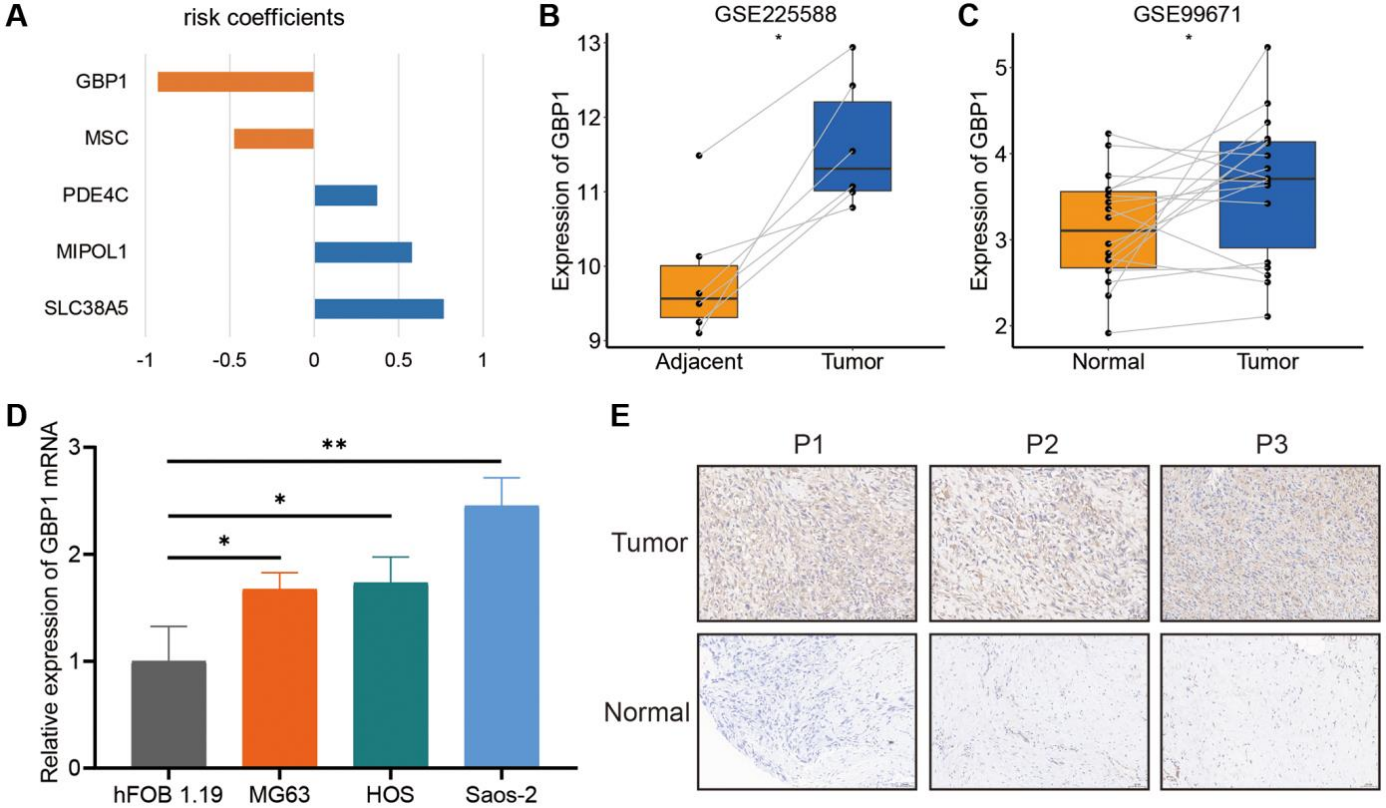
**The expression levels of GBP1.** (**A**) The coefficients of the five model genes. (**B**, **C**) The GBP1 expression level in osteosarcoma and non-tumoral paired samples, based on the GSE99671 and GSE225588 cohort. (**D**) The qRT-PCR result of GBP1 in hFOB 1.19, MG63, HOS, Saos-2 cell lines. (**E**) The expressions of GBP1 in tumor and adjacent normal tissues. ^*^P < 0.05, ^**^P < 0.01 and ^***^P < 0.001.

The impact of GBP1 on the score was found to be the greatest, making it the preferred subject for further investigation (Figure 7A). Analysis of the GSE225588 and GSE99671 datasets revealed a considerable upregulation of GBP1 expression in osteosarcoma samples (Figure 7B, 7C). Furthermore, the quantification of GBP1 expression was performed in both osteosarcoma and osteoblasts cell lines. The results showed a significant increase in GBP1 expression levels in osteosarcoma cells compared to osteoblasts (Figure 7D). In addition, high expression of GBP1 was detected in tumor tissues using immunohistochemical staining (Figure 7E).

### Pan-cancer analysis of GBP1

**Figure 8:**
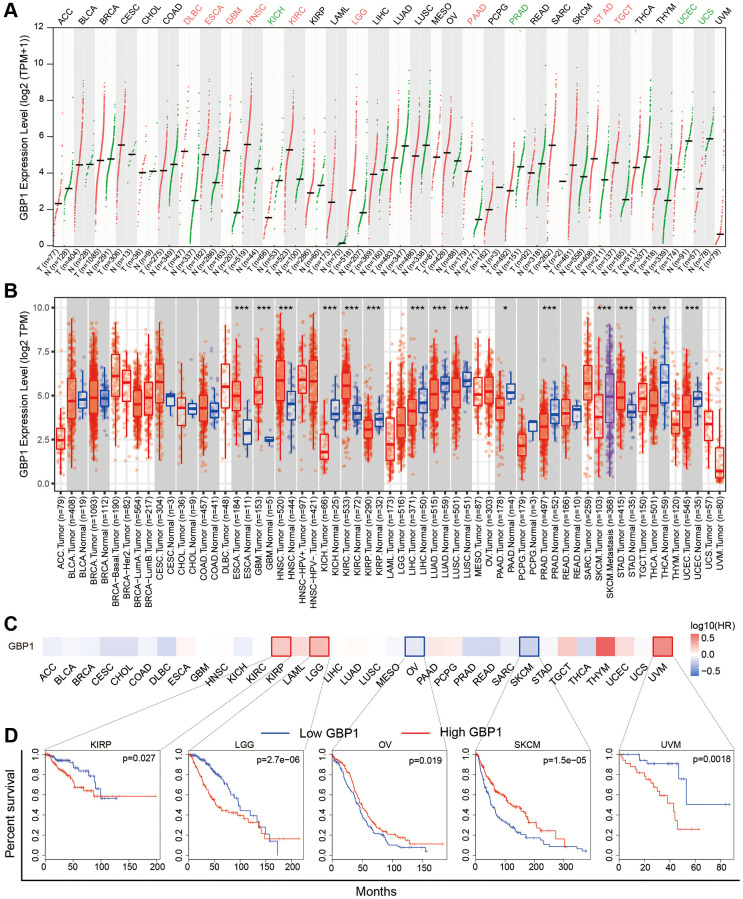
**Expression and significance of GBP1 in Pan-caner.** (**A**) Pan-cancer analysis of GBP1 expression based on the GEPIA2. (**B**) Pan-cancer analysis of GBP1 expression based on TIMER. (**C**) The survival map with positive results were presented. (**D**) Kaplan-Meier survival curves for overall survival rate over TCGA cancer types. ^*^P < 0.05, ^**^P < 0.01 and ^***^P < 0.001.

An extensive investigation into the expression pattern of GBP1 in pan-cancer was conducted. The findings indicated a high prevalence of GBP1 expression in numerous tumors, such as those found in the esophageal cancer, glioblastoma, kidney clear cell carcinoma, and stomach. Conversely, reduced expression was observed in kidney chromophobe, prostate cancer, and endometrioid cancer, underscoring the importance of GBP1 as a key biomarker across a wide range of cancers (Figure 8A, 8B). Additionally, we conducted a pan-cancer analysis to examine the influence of GBP1 on cancer prognosis. The results from Kaplan-Meier analysis indicated that GBP1 could act as a risk factor for ovarian cancer and skin cutaneous melanoma, while it may serve as a protective factor for lower grade glioma, kidney renal papillary cell carcinoma, and uveal melanoma (Figure 8C, 8D). These discoveries highlight the considerable prognostic implications of GBP1 in a pan-cancer.

## DISCUSSION

Despite substantial advancements in diagnosing and treating osteosarcoma, the global mortality rate associated with this disease continues to be alarming [31, 32]. The IE enables neoplastic cells to persist and proliferate despite the vigilant scrutiny exerted by the immune system [33, 34]. Therefore, our study aimed to comprehensively evaluate the prognosis of IEGs in osteosarcoma and explore potential biological differences. The results of this study will enhance our understanding of the function of IEGs in osteosarcoma and help design more effective treatment strategies.

Based on 182 IEGs, consensus clustering analysis identified two distinct osteosarcoma clusters in the present study. These two clusters displayed significant differences in prognosis and immune cluster. IEGcluster A demonstrated superior survival advantages and increased immune cell infiltration compared to IEGcluster B. Prior studies have emphasized the critical role of TME in tumor progression. Based on these findings, a higher level of immune infiltration may be responsible for the better prognosis of IEGcluster A. Furthermore, DEGs, associated with immune receptor and GTPase activity, were identified between the two clusters and recognized as IEGs. Consistent clustering analysis of DEGs led to identifying two gene clusters exhibiting significant prognosis differences. These findings further validate the existence of two distinct IEG clusters and that the collective effects of multiple IEGs are integral to immune infiltration.

Given the heterogeneous nature of tumors, an IE score system was developed to quantify individual patients, thus offering a more precise direction for personalized treatment. Immunological subtypes, characterized by strong immune infiltration, exhibited lower IE scores. The results underscored the potential of the IE score as a valuable tool for assessing the immune evasion cluster and evaluating immune infiltration in individual osteosarcoma patients. The connection between IEGs and tumor metastasis is well-established, so the IE score differences in patients across various clinical clusters were examined. The findings suggest that the IE score can be valuable for evaluating patient metastasis. An independent prognostic indicator for osteosarcoma can be determined by a comprehensive analysis of the IE score.

GBP1, a distinctive large GTPase enzyme, regulates cellular reactions to infection, inflammation, and environmental stress [35–38]. Its role varies across different cancer types [39, 40]. Cytokine stimulation in tumor cells leads to the release of soluble GBP1 both *in vivo* and *in vitro*, suggesting a potential antitumor effect in ovarian cancer [41]. These findings align with the results from our pan-cancer analysis. Moreover, GBP1 has been associated with promoting lymph node metastasis in esophageal squamous cell carcinoma [42]. In line with recent research findings and our results, GBP1 seems to act as a protective factor in osteosarcoma [43, 44]. An examination of multiple public databases revealed an upregulation of GBP1 in various tumors, including osteosarcoma. RT-qPCR further confirmed high GBP1 expression in osteosarcoma cell lines. These observations indicate the potential of GBP1 as a biomarker for osteosarcoma.

However, there are some limitations to this study. Firstly, it relies on publicly available datasets, which might introduce potential biases. For more accurate predictions, larger sample sizes need to be used in the future. Secondly, there is a requirement for more comprehensive clinical data to evaluate the correlation between scores and clinical characteristics. Finally, more datasets are needed to validate the differential expression of five IEGs.

## CONCLUSION

In this investigation, two distinct clusters of patients were identified based on IEGs, and an assessment of immune infiltration characteristics was conducted by systematically comparing various clusters. The IE score was further established to evaluate clinical features and immune infiltration status in individual osteosarcoma patients. Ultimately, a biomarker was discovered and validated. This research offers novel insights for identifying new tumor clusters in osteosarcoma, directing personalized and specific therapies, and enhancing patient responses to immunotherapy.

## Supplementary Materials

Supplementary Table 1

Supplementary Table 2
