# Supplementary material for: Molecular characterization of cancer-intrinsic immune evasion genes indicates prognosis and tumour microenvironment infiltration in osteosarcoma
Source: Aging (Albany NY). 2023 Oct 4;15(19):10272–90. doi: 10.18632/aging.205074 (PMC10599718; doi:10.18632/aging.205074)
Supplement: Supplementary Table 2 [file aging-15-205074-s002.pdf]

SUPPLEMENTARY TABLE

Supplementary Table 2. Primers used in this study.

Gene	Forward primer 5′–3′	Reverse primer 5′–3′
GBP1	TATTGCCCACTATGAACAGCAGAT	TAGCTGGGCCGCTAACTCC
